# An improved approach for measuring immersion freezing in large droplets over a wide temperature range

**DOI:** 10.1038/srep32930

**Published:** 2016-09-06

**Authors:** Yutaka Tobo

**Affiliations:** 1 National Institute of Polar Research, Tachikawa, Tokyo 190-8518, Japan; 2 Department of Polar Science, School of Multidisciplinary Sciences, SOKENDAI (The Graduate University for Advanced Studies), Tachikawa, Tokyo 190-8518, Japan

## Abstract

Immersion freezing (ice nucleation by particles immersed in supercooled water) is a key process for forming ice in mixed-phase clouds. Immersion freezing experiments with particles in microliter-sized (millimeter-sized) water droplets are often applied to detecting very small numbers of ice nucleating particles (INPs). However, the application of such large droplets remains confined to the detection of INPs active at temperatures much higher than the homogeneous freezing limit, because of artifacts related to freezing of water droplets without added INPs at temperatures of −25 °C or higher on a supporting substrate. Here I report a method for measuring immersion freezing in super-microliter-sized droplets over a wide temperature range. To reduce possible artifacts, droplets are pipetted onto a thin layer of Vaseline and cooled in a clean booth. In the Cryogenic Refrigerator Applied to Freezing Test (CRAFT) system, freezing of pure (Milli-Q) water droplets are limited at temperatures above −30 °C. An intercomparison of various techniques for immersion freezing experiments with reference particles (Snomax and illite NX) demonstrates that despite the use of relatively large droplets, the CRAFT setup allows for evaluating the immersion freezing activity of the particles over almost the entire temperature range (about −30 °C to 0 °C) relevant for mixed-phase cloud formation.

Mixed-phase clouds, composed of both ice crystals and supercooled cloud droplets, are typically formed under conditions above water saturation at temperatures between about −36 °C and 0 °C. It is thought that although heterogeneous ice nucleation processes can occur via several pathways, immersion freezing (ice nucleation by solid particles immersed in supercooled water) is the dominant process for ice nucleation in mixed-phase clouds and only a subset of cloud droplets containing ice nucleating particles (INPs; certain particles capable of nucleating ice) can freeze heterogeneously^1^,^2^,^3^. Subsequently, ice multiplication (secondary ice production) may also proceed in mixed-phase clouds^1^,^4^. Since ice is thermodynamically more stable than supercooled water at temperatures below 0 °C, it is expected that ice crystals in mixed-phase clouds can grow in size more efficiently than ambient cloud droplets^5^,^6^, thus influencing ice-induced precipitation, cloud lifetime and radiative properties.

Measuring the number concentrations of INPs active under mixed-phase cloud conditions still remains challenging, because the values vary over many orders of magnitude (approximately, 10^−6^ to 10^4^ L^−1^) depending on temperatures, geophysical locations and atmospheric conditions^7^,^8^,^9^. Techniques for freezing experiments with single particles coated with supercooled water (cloud-sized droplets in picoliter-/nanoliter-size ranges) and suspended in gas (for example, controlled expansion cloud-simulation chamber^10^,^11^,^12^, continuous flow diffusion chamber^7^,^9^,^13^,^14^,^15^,^16^, continuous flow mixing chamber^17^, laminar flow tube^18^, electrodynamic balance levitator^19^) have been applied to investigate their ice nucleating ability under mixed-phase cloud conditions. However, the use of these techniques is limited to the detection of relatively high numbers of INPs (typically, >1 L^−1^). An alternative technique involves freezing experiments with particles immersed in supercooled water droplets on a cold stage. In particular, sub-/super-microliter-sized droplets (millimeter-sized droplets which are much larger than cloud-sized droplets) containing multiple particles have been used for the detection of rare, but highly efficient INPs^8^,^20^,^21^,^22^,^23^,^24^,^25^,^26^,^27^,^28^. A major problem in previous cold-stage-based freezing experiments with sub-/super-microliter-sized water droplets is that some of the water droplets without any added INPs start to freeze at temperatures of −25 °C or higher and then the majority of them freeze before reaching −30 °C. This is probably because larger water droplets are more easily influenced by contamination in the droplets or on the supporting substrates, resulting in their freezing at temperatures much warmer than the homogeneous freezing limit^3^. Thus, it has been thought that cold-stage-based freezing experiments with sub-/super-microliter-sized droplets are only applicable to measuring immersion freezing at relatively warm temperatures under mixed-phase cloud conditions.

Here, I set out to test whether a few simple modifications to cold-stage-based freezing techniques could be useful in reducing possible artifacts related to freezing of large pure water droplets and in evaluating the ice nucleating ability of particles immersed in the droplets over a wide temperature range relevant for mixed-phase cloud formation.

## Methods

### Experimental setup

**Figure 1:**
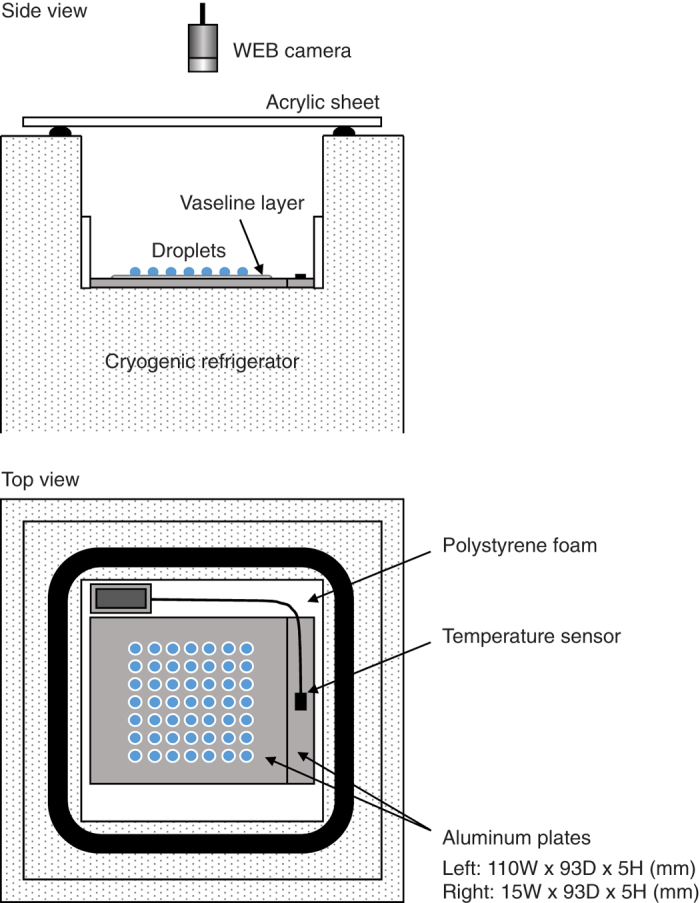
Schematics of the Cryogenic Refrigerator Applied to Freezing Test (CRAFT) setup.

The overview of the setup for the freezing experiment system, termed a Cryogenic Refrigerator Applied to Freezing Test (CRAFT), is illustrated in Fig. 1. After spreading a thin layer of Vaseline (petroleum jelly, a semi-solid mixture of hydrocarbons) on the surface of an aluminum plate using a plastic spatula, droplets were pipetted onto the plate with an Eppendorf micropipette. These procedures were performed in a clean bench to avoid contamination of the droplets by airborne particles. In the clean bench, the number concentrations of airborne particles with diameters larger than 0.3 μm measured using an optical particle counter were less than 0.1 L^−1^. The plate was set and cooled on the stage of a portable cryogenic refrigerator (CRYO PORTER, Model CS-80CP, Scinics Corporation, Tokyo, Japan). The cryogenic refrigerator is a Stirling engine-based device that can control the temperature of the stage between −80 °C and 50 °C. The uncertainty of the temperature is ±0.2 °C. It is also possible to create a program to regulate the cooling/heating ratio via an external keypad controller. The temperature of the plate was measured using a single temperature sensor attached on the surface of the aluminum plate (note that the temperature uniformity on the plate was confirmed in preliminary tests with multiple temperature sensors). Each freezing experiment was monitored by a conventional WEB camera. Based on the video image analysis, the number fractions of droplets frozen (*f*_frozen_) and unfrozen (*f*_unfrozen_) were counted every 0.5 °C. It is important to note that both the clean bench and the cryogenic refrigerator were installed in a clean booth. In the clean booth, the number concentrations of airborne particles with diameters larger than 0.3 μm were small (typically, less than 10 L^−1^), but the top part of the refrigerator was covered with an acrylic sheet to further reduce the possibility of contamination of the droplets in the booth during each freezing experiment. No purging with dry gas (for example, N_2_ gas) was applied in the CRAFT setup. The room temperature and relative humidity were approximately 20 °C and 40%RH, respectively.

### Freezing experiments

The CRAFT setup was used to conduct freezing experiments with Milli-Q purified water (≥18 MΩ cm) droplets and droplets containing Snomax (biological materials from the bacterium *Pseudomonas syringae*) or illite NX (illite-rich mineral dusts). Both Snomax and illite NX have been used as reference particles for intercomparison studies on immersion freezing experiments performed in the framework of the Ice Nuclei research UnIT (INUIT) project (see refs 29 and 30 for details). In this study, the initial mass concentration of Snomax or illite NX particles suspended in Milli-Q water was adjusted to 1 mg/mL and then serially diluted 100-fold with Milli-Q water. Although it is potentially possible to use 10 μL or larger droplets for cold-stage-based freezing experiments^21^,^22^,^23^,^24^,^31^, it is expected that a cooling rate of <1 °C/min is required to effectively minimize temperature gradients between the cold stage and droplet and/or within the droplet and that the use of such a low cooling rate promotes evaporation of water from droplets. In this study, 49 droplets with a volume of 5 μL were used for all the freezing experiments and the temperature was lowered at a cooling rate of 1 °C/min until all the droplets froze. In this setup, no measureable shrinking of droplets induced by evaporation was confirmed at the end of each freezing experiment. The results of control experiments with Milli-Q water droplets are presented in the Results section.

### Data analysis

By assuming that ice nucleation by particles immersed in supercooled droplets can be regarded as a temperature-dependent and time-independent process, the cumulative number of ice nucleation active sites per unit volume of water (*K*) at a given temperature (*T*) can be expressed as^3^,^20^:


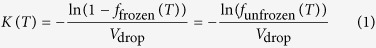


where *V*_drop_ is the volume of a droplet (=5 μL). If the mass of particles per unit volume of water is known, then the ice nucleation active site density per unit mass (*n*_m_) can also be derived:


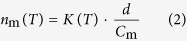


where *C*_m_ is the mass concentration of the particles in the initial suspension (=1 mg/mL) and *d* is the dilution ratio of the suspension relative to *C*_m_. The *n*_m_ values for Snomax and illite NX particles were calculated by analyzing the data obtained from the freezing experiments using the CRAFT. If the surface-to-mass ratio of the particles (*S*_total_/*M*_total_) is known, then the ice nucleation active site density per unit surface area (*n*_s_) can be estimated by the following equation^30^:


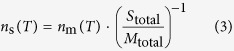


In this study, the *n*_s_ values for illite NX particles were obtained assuming that the *S*_total_/*M*_total_ value is 6.54 m^2^ g^−1^ (geometric surface area-based value) for illite NX^30^.

## Results

### Control experiments

**Figure 2:**
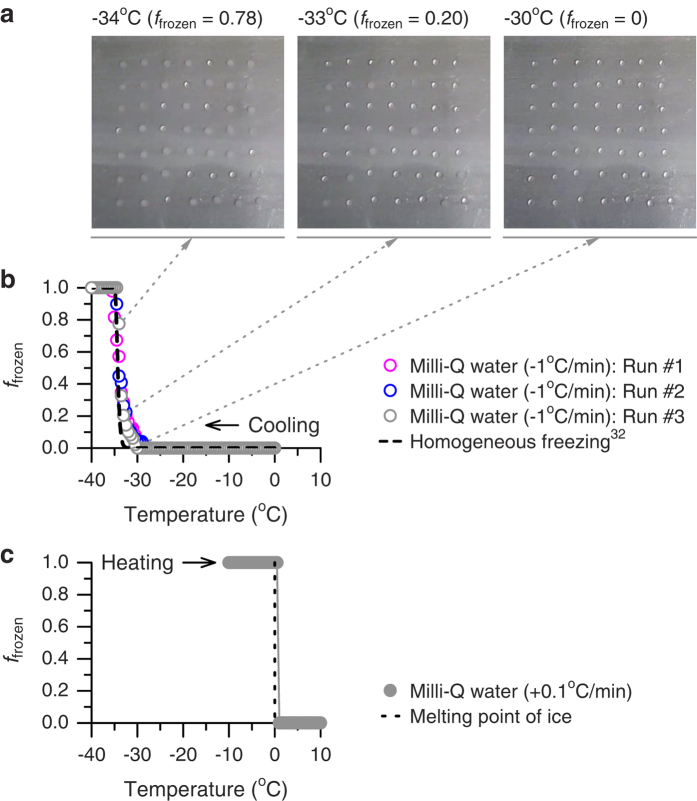
Experiments with Milli-Q water droplets performed using the CRAFT. (**a**) Images of the droplets during a freezing experiment. (**b**) Fraction of the droplets frozen (*f*_frozen_) as a function of temperature during freezing experiments. The data were obtained from three different experiments. Also shown is a classical nucleation theory-based parameterization for homogeneous freezing of 5 μL pure water droplets with a cooling rate of 1 °C/min (ref. 32). (**c**) Fraction of the droplets frozen as a function of temperature during a melting experiment with a heating rate of 0.1 °C/min.

Earlier studies have reported practical difficulties when applying sub-/super-microliter-sized droplets to freezing experiments on a cold substrate^8^,^20^,^21^,^22^,^23^,^24^,^25^,^26^,^27^,^28^, because of possible artifacts causing freezing of pure water droplets at temperatures much warmer than the temperature expected for homogeneous freezing. To evaluate possible artifacts related to the use of super-microliter-sized droplets in the CRAFT setup, freezing experiments with Milli-Q water droplets with a volume of 5 μL were performed with a cooling rate of 1 °C/min. The results show that despite the use of super-microliter-sized droplets, none of the droplets freeze before reaching temperatures of about −30 °C to −28 °C (Fig. 2a,b). The results also show that the fraction of the droplets frozen dramatically increases at around the onset temperature of homogenous freezing of 5 μL pure water droplets (within −35 °C to −33 °C) predicted based on a classical nucleation theory^32^. Note that the homogeneous freezing temperature of 5 μL water droplets is expected to be about 3–5 °C higher than that of picoliter-/nanoliter-sized water droplets (Supplementary Fig. S1). In addition, the melting temperature of the frozen water droplets was measured during heating at 0.1 °C/min (Fig. 2c). The visually apparent melting temperature range measured here (0.5 °C to 1 °C) is only slightly higher than the melting point of water ice and that of 1 μL frozen droplets measured by Whale *et al*.^27^ (−0.1 °C to 0.6 °C). Thus, the present results indicate that freezing/melting behaviors of 5 μL Milli-Q water droplets in the CRAFT setup are reasonably consistent with the literature values and that the temperature difference between the cold stage and droplets is small.

### Freezing experiments with reference particles

**Figure 3:**
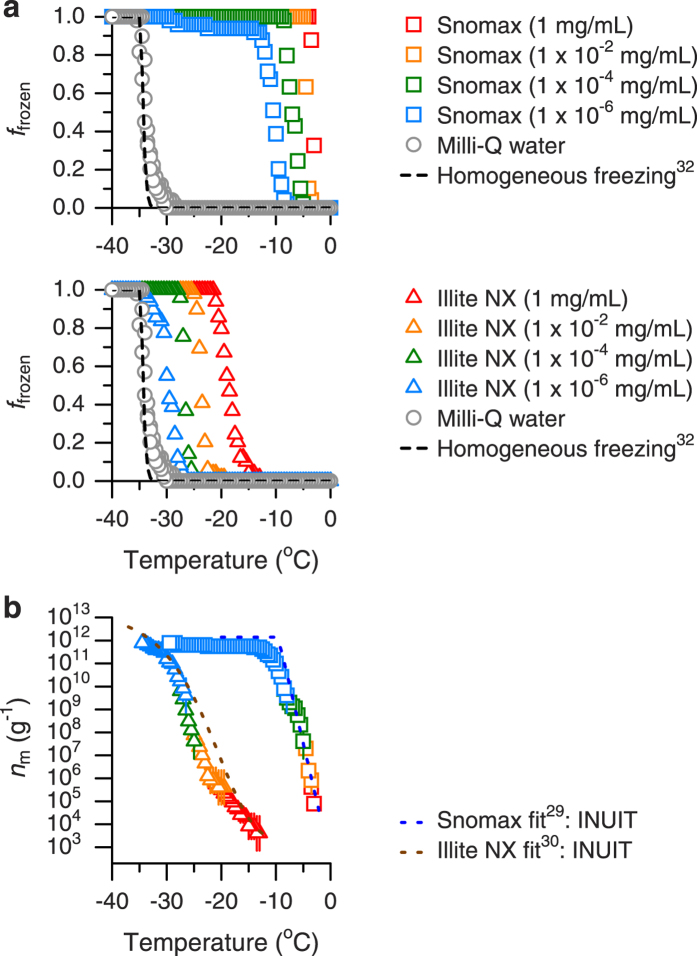
Freezing experiments with Snomax or illite NX particles immersed in water droplets performed using the CRAFT. (**a**) Fraction of the droplets frozen as a function of temperature. The mass concentrations in water are between 1 × 10^6^ and 1 mg/mL. Also shown are the data on Milli-Q water droplets (Runs #1, #2 and #3 in Fig. 2b) and a classical nucleation theory-based parameterization for homogeneous freezing of 5 μL pure water droplets with a cooling rate of 1 °C/min (ref. 32). (**b**) Ice nucleation active site densities per unit mass (*n*_m_) for Snomax and illite NX. The *n*_m_ parameterizations designed in the INUIT project^29^,^30^ are also shown. Error bars represent the 95% confidence intervals.

The performance of the CRAFT setup was further evaluated with the use of droplets containing reference particles (Snomax and illite NX particles). Their mass concentrations in Milli-Q water were regulated between 1 × 10^−6^ and 1 mg/mL, corresponding to a total mass between 5 pg and 5 μg per droplet, respectively. The results of these experiments are summarized in Fig. 3. The freezing temperatures of droplets containing Snomax or illite NX particles are clearly warmer than those of pure Milli-Q water droplets (Fig. 3a). The majority of the droplets containing Snomax or illite NX particles freeze at temperatures between about −13 °C and −3 °C and between about −33 °C and −13 °C, respectively, and their freezing temperatures are lowered by decreasing the mass concentrations. Based on these experimental results, the ice nucleation active site densities per unit mass for Snomax and illite NX particles in the immersion mode were determined as a function of temperature (Fig. 3b). The results show that the *n*_m_ values for Snomax particles are much higher than those for illite NX particles at temperatures warmer than about −30 °C, thus indicating that Snomax particles are much more efficient INPs at the warmer temperatures. The ice nucleating abilities of both Snomax and illite NX particles in the immersion mode have been well studied using various existing measurement techniques within the framework of the INUIT project^29^,^30^. As illustrated in Fig. 3b, the *n*_m_ values determined using the CRAFT are in reasonably good agreement with the *n*_m_ parameterizations for Snomax (equation (6) in ref. 29) and illite NX (equation (3) in ref. 30 combined with the equation “All (log)” in Table 3 in ref. 30) designed from the data collected in the INUIT project.

**Figure 4:**
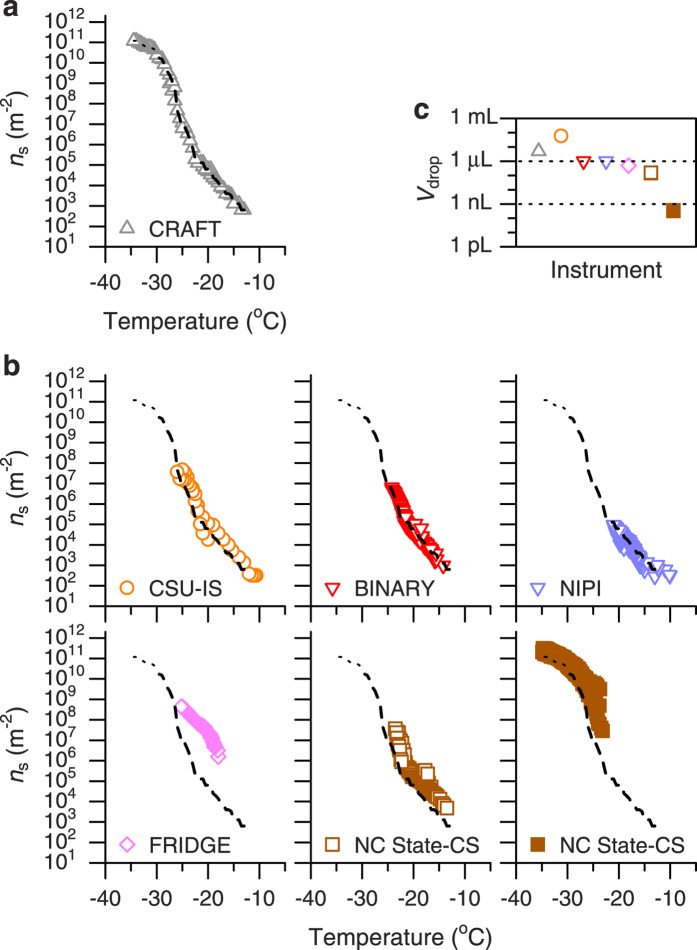
Intercomparison of cold-stage-based freezing experiments with illite NX particles immersed in water droplets. (**a,b**) Ice nucleation active site densities per unit surface area (*n*_s_) obtained using the CRAFT and a variety of instruments involved in the INUIT project^30^. Dotted and dashed curves are from the data at temperatures below and above −30 °C in (**a**), respectively. (**c**) Volume of the droplets (*V*_drop_) used for each experiment.

The ice nucleating ability of illite NX particles measured here was further compared with the results from other measurement techniques on the basis of the ice nucleation active site densities per unit surface area. Figure 4 shows the *n*_s_ values determined using the CRAFT (Fig. 4a) and other cold-stage-based droplet freezing techniques (Fig. 4b) including the Colorado State University Ice Spectrometer^9^,^24^ (CSU-IS), the Bielefeld Ice Nucleation ARraY^25^ (BINARY), the Nucleation by Immersed Particle Instrument^26^,^27^ (NIPI), the FRankfurt Ice Deposition freezinG Experiment^30^,^33^ (FRIDGE) and the North Carolina State Cold Stage^8^,^28^ (NC State-CS). The sizes of droplets used for each experiment are summarized in Fig. 4c. The results show that the *n*_s_ values obtained using the CRAFT are consistent with those from other similar techniques except the FRIDGE (the cause of the higher *n*_s_ values at temperatures warmer than about −25 °C indicated by the FRIDGE data is uncertain^30^). However, the data obtained using the CSU-IS, BINARY and NIPI are limited to a relatively warm temperature range and the NS State-CS data also indicate a similar result when using sub-microliter-sized droplets. This is mainly due to artifacts related to the activation of freezing of water droplets without any added INPs at temperatures much warmer than −30 °C. In the NS State-CS setup, however, the influence of such freezing artifacts is greatly reduced when using nearly cloud-sized droplets^28^ (droplets smaller than 1 nL in volume). It is noteworthy that despite the use of super-microliter-sized droplets, the *n*_s_ values derived from the CRAFT are not limited by freezing artifacts over a wide temperature range down to about −30 °C (Figs 2b and 3a). As shown in Fig. 4, the *n*_s_ values obtained using 5 μL droplets in the CRAFT setup are reasonably consistent with those determined using droplets smaller than 1 nL in the NS State-CS setup in the temperature range colder than about −25 °C.

**Figure 5:**
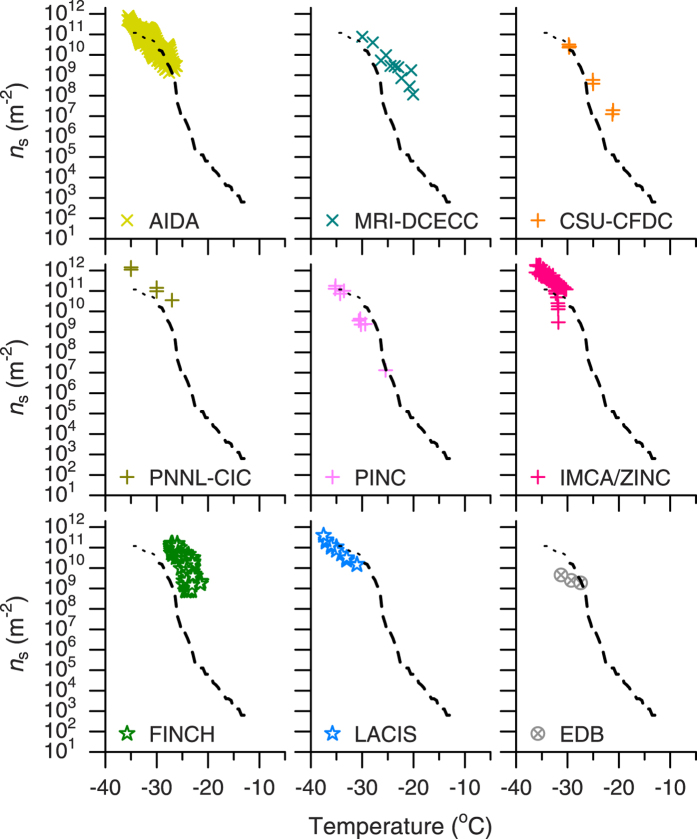
Same as Fig. 4b, but for freezing experiments with single illite NX particles coated with supercooled water and suspended in gas.

Comparisons with the *n*_s_ values of illite NX particles obtained using freezing experiments with single particles coated with supercooled water (cloud-sized droplets) and suspended in gas are presented in Fig. 5. The experimental techniques include the Aerosol Interactions and Dynamics in the Atmosphere (AIDA) chamber^10^,^11^, the Meteorological Research Institute Dynamically Controlled Expansion Cloud-simulation Chamber^12^ (MRI-DCECC), the Colorado State University Continuous Flow Diffusion Chamber^7^,^9^,^13^ (CSU-CFDC), the Pacific Northwest National Laboratory Compact Ice Chamber^14^ (PNNL-CIC), the Portable Ice Nucleation Chamber^15^ (PINC), the Immersion Mode Cooling chAmber combined with Zurich Ice Nucleation Chamber^16^ (IMCA/ZINC), the Fast Ice Nucleus CHamber^17^ (FINCH), the Leipzig Aerosol Cloud Interaction Simulator^18^ (LACIS) and an electrodynamic balance (EDB) levitator^19^. Note that the techniques listed here have been applied to the evaluation of the ice nucleating ability of dry-dispersed particles in the immersion mode^30^. Since these techniques cannot typically count very small numbers of INPs^30^, the data presented in Fig. 5 are confined to the ranges of relatively high *n*_s_ values. Nevertheless, the *n*_s_ values determined using these techniques are approximately consistent with those obtained using the CRAFT setup at temperatures around −30 °C. The FINCH data show the highest *n*_s_ values in the temperature range between about −27 °C and −22 °C. In this regard, Hiranuma *et al*.^30^ noted that the FINCH might overestimate the *n*_s_ values due to uncertainties in the INP number concentration, *S*_total_ or temperature data. Hiranuma *et al*.^30^ also suggested that the higher *n*_s_ values derived from the MRI-DCECC at warmer temperatures might be related in part to the detection limit of a particle counter used to measure ice crystals.

## Discussion

The results presented here demonstrate that the simple CRAFT setup allows the observation of immersion freezing to nearly the homogeneous freezing limit of pure water droplets in the super-microliter-size range. In addition, freezing experiments with the droplets containing two different types of reference particles (Snomax and illite NX) indicate that the CRAFT setup is useful in evaluating their ice nucleating abilities in the immersion mode over a wide temperature range relevant for mixed-phase cloud formation by simply adjusting the mass concentrations in the droplets and not the droplet volumes. This work yields important progress in conducting cold-stage-based freezing experiments with relatively large droplets, since it has been thought that quantifying immersion freezing on a cold stage at temperatures down to nearly the homogeneous freezing limit is only possible when using cloud-sized small droplets^28^,^32^. Freezing experiments with super-microliter-sized droplets containing multiple particles have the advantage of being able to detect very low numbers of INPs. Another advantage of using such droplets instead of cloud-sized droplets is their ease of handling; the former droplets can be easily prepared by pipetting and the freezing behaviors of the droplets are confirmable without optical magnification.

A major difference between the CRAFT and other similar cold-stage-based techniques might be the difference in materials used as a cold substrate. In the CRAFT system, droplets were placed on a semi-solid hydrophobic surface (a thin Vaseline layer). It is expected that the Vaseline coating on a cold stage can play a key role in preventing the influence of frost growth related to freezing of neighboring droplets as well as water vapor deposition on the surface of the substrate^34^,^35^. On the other hand, many other studies have employed solid hydrophobic substrates; for example, 96-well microtiter plates in the CSU-IS^9^,^24^ and hydrophobized glass/silicon substrates in the BINARY^25^, NIPI^26^,^27^, FRIDGE^30^,^33^ and NC State-CS^8^,^28^. In addition, these studies have tried to reduce possible frost growth on the substrates by conducting freezing experiments in dry N_2_ purge gas or oil; however, all these experiments have indicated that sub-/super-microliter-sized pure water droplets can freeze at temperatures some tens of degrees above the homogeneous freezing threshold. Considering that neither dry purge gas nor oil was used in the CRAFT, these treatments might not necessarily be important when preparing droplets on a semi-solid hydrophobic surface like the Vaseline coating. It is also important to note that all key procedures, such as pipetting and freezing of droplets, were conducted within a clean bench/booth. The use of the clean space for all these procedures appears to be one of the most important points to reduce possible contamination of droplets in laboratory air.

Freezing experiments with super-microliter-sized droplets in the CRAFT system proved to be a simple method to measure INPs over wide ranges of temperature and particle concentration under mixed-phase cloud conditions. It is thought that cloud-chamber-based freezing experiments are typically not a useful approach to detecting very low numbers of INPs; hence, the available data are limited to the conditions of relatively high *n*_s_ values in a colder temperature range. Thus, cold-stage-based techniques fill an important need. However, some recent studies have pointed out that cold-stage-based droplet freezing experiments sometimes underestimate the number of INPs, as compared with cloud-chamber-based freezing experiments, especially at warmer temperatures^30^,^36^. In this regard, the reduction of the available surface area for ice nucleation (affecting the apparent *n*_s_ values) might occur in cold-stage-based experiments if large amounts of particles suspended in the droplets aggregate and/or settle out. The size dependence of the settling distances of Snomax and illite NX particles in water was estimated assuming that they are spherical particles with a density of 1.35 and 2.65 g/cm^3^, respectively^29^,^30^ (Supplementary Fig. S2). Given that the diameters of Snomax and illite NX particles used here are mainly distributed between about 0.1 and 1 μm (refs 29 and 30), it is expected that the settling distances of these particles during each freezing experiment with a cooling rate of 1 °C/min (the duration of each experiment is less than 60 minutes) are shorter than the diameter of 5 μL water droplets (a few micrometers). It should also be noted here that the visually apparent sedimentation of the particles was not observed even in the highly-concentrated initial suspensions (=1 mg/mL). These results imply that the majority of Snomax and illite NX particles used here were still suspended in the water droplets during freezing experiments. Nevertheless, further validation studies will be necessary to verify the full utility of cold-stage-based droplet freezing techniques including the CRAFT.

## Additional Information

**How to cite this article**: Tobo, Y. An improved approach for measuring immersion freezing in large droplets over a wide temperature range. *Sci. Rep.* **6**, 32930; doi: 10.1038/srep32930 (2016).

## Supplementary Material

Supplementary Information
