# Supplementary material for: An improved approach for measuring immersion freezing in large droplets over a wide temperature range
Source: Sci Rep. 2016 Sep 6;6:32930. doi: 10.1038/srep32930 (PMC5011777; doi:10.1038/srep32930)
Supplement: Supplementary Information [file srep32930-s1.pdf]

Supplementary Information

An improved approach for measuring immersion freezing in large droplets over a wide temperature range

Yutaka Tobo[1,2,*]

[1]National Institute of Polar Research, Tachikawa, Tokyo 190-8518, Japan
[2]Department of Polar Science, School of Multidisciplinary Sciences, SOKENDAI (The Graduate University for Advanced Studies), Tachikawa, Tokyo 190-8518, Japan

[*]tobo.yutaka@nipr.ac.jp

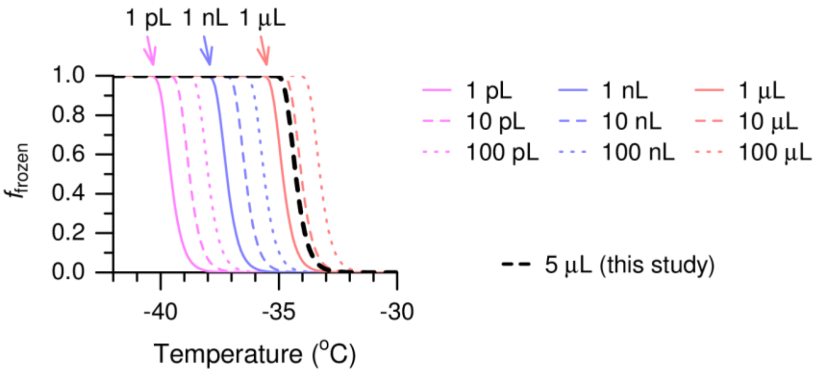

Supplementary Figure S1. Fraction of droplets frozen as a function of temperature for pure water droplets of different volumes. These values are obtained using a classical nucleation theory-based parameterization for homogeneous freezing with a cooling rate of 1°C/min (ref. 32).

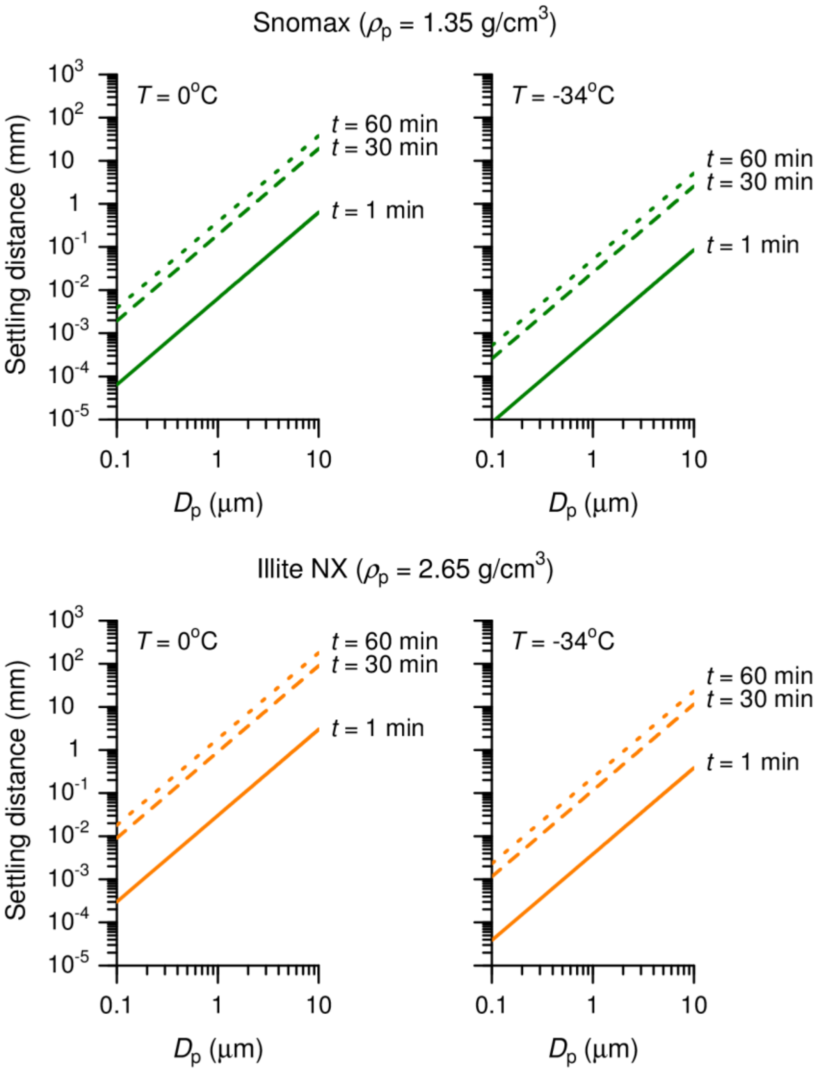

Supplementary Figure S2. Size dependence of the settling distances of Snomax or illite NX particles in water at temperatures of 0°C and -34°C. The settling distances after 1, 30 and 60 minutes are estimated based on the terminal setting velocities of the particles (see Supplementary Methods).

Supplementary Methods

According to Stokes' law, the terminal settling velocity (v) of small spherical particles in water is expressed as:

$$v = \frac{(\rho_p - \rho_w) \cdot g}{18\eta_w} \cdot {D_p}^2 \tag{S1}$$

where η_w is the viscosity of water, ρ_w is the density of water, ρ_p is the density of particles, D_p is the particle diameter, and g is the gravitational acceleration (= 9.80665 m/s^2). In the calculations, it is assumed that the η_w and ρ_w values are temperature dependent parameters in the temperature range between -34°C and 0°C (refs 37 and 38) and that the ρ_p values of Snomax and illite NX particles are ~1.35 g/cm^3 and ~2.65 g/cm^3, respectively[29,30]. The settling distances of Snomax and illite NX particles in water at 0°C and -34°C are then estimated using the v values. Supplementary Figure S2 illustrates the settling distances after t minutes (t = 1, 30 and 60). Given that the diameters of Snomax and illite NX particles used here are mainly distributed between about 0.1 and 1 μm (refs 29 and 30), it is expected that the majority of them are suspended in 5 μL water droplets (a few micrometers in diameter) even at the end of each freezing experiment (i.e., the settling distances of the particles are shorter than the diameter of the droplets).

References

37. Dehaoui, A., Issenmann, B. & Caupin, F. Viscosity of deeply supercooled water and its coupling to molecular diffusion. *Proc. Natl Acad. Soc. USA* **112**, 12020-12025 (2015).

38. Hare, D. E. & Sorensen, C. M. The density of supercooled water. II. bulk samples cooled to the homogeneous nucleation limit. *J. Chem. Phys.* **87**, 4840-4845 (1987).
